# Supplementary material for: The landscape of receptor-mediated precision cancer combination therapy via a single-cell perspective
Source: Nat Commun. 2022 Mar 25;13:1613. doi: 10.1038/s41467-022-29154-2 (PMC8956718; doi:10.1038/s41467-022-29154-2)
Supplement: Supplementary file 2 — Reporting Summary [file 41467_2022_29154_MOESM2_ESM.pdf]

nature research

Corresponding author(s): Alejandro Schaffer and Eytan Ruppin

Last updated by author(s): February 10, 2022

Reporting Summary

Nature Research wishes to improve the reproducibility of the work that we publish. This form provides structure for consistency and transparency in reporting. For further information on Nature Research policies, see our Editorial Policies and the Editorial Policy Checklist.

Statistics

For all statistical analyses, confirm that the following items are present in the figure legend, table legend, main text, or Methods section.

n/a	Confirmed	
☐	☒	The exact sample size (*n*) for each experimental group/condition, given as a discrete number and unit of measurement
☐	☒	A statement on whether measurements were taken from distinct samples or whether the same sample was measured repeatedly
☒	☐	The statistical test(s) used AND whether they are one- or two-sided<br>*Only common tests should be described solely by name; describe more complex techniques in the Methods section.*
☐	☒	A description of all covariates tested
☐	☒	A description of any assumptions or corrections, such as tests of normality and adjustment for multiple comparisons
☐	☒	A full description of the statistical parameters including central tendency (e.g. means) or other basic estimates (e.g. regression coefficient) AND variation (e.g. standard deviation) or associated estimates of uncertainty (e.g. confidence intervals)
☒	☐	For null hypothesis testing, the test statistic (e.g. *F*, *t*, *r*) with confidence intervals, effect sizes, degrees of freedom and *P* value noted<br>*Give P values as exact values whenever suitable.*
☒	☐	For Bayesian analysis, information on the choice of priors and Markov chain Monte Carlo settings
☐	☒	For hierarchical and complex designs, identification of the appropriate level for tests and full reporting of outcomes
☒	☐	Estimates of effect sizes (e.g. Cohen's *d*, Pearson's *r*), indicating how they were calculated

Our web collection on statistics for biologists contains articles on many of the points above.

Software and code

Policy information about availability of computer code

Data collection	Primary data we analyzed are available in GEO or ArrayExpress, but in some cases we had to contact the submitting authors to provide metadata that are not in the public repositories. Because the public data were not formatted homogeneously, we had to write some one-off programs to reformat data sets into a homogenous format expected by our software MadHitter. The format is explained in our software documentation and examples of properly formatted data are included with the MadHitter software distribution. The lightly processed input data are available at https://ftp.ncbi.nlm.nih.gov/pub/catSMA/MadHitter_data. The input data processed for this study have also been deposited in Zenodo and can be located via the doi 10.5281/zenodo.6038684. All of this information and more is now in the Data Avaialbility subsecton near the end of Methods.
Data analysis	The focus of the paper is the new software MadHitter, which is freely available via https://github.com/ruppinlab/madhitter. We also deposited the current version in Zeno with doi doi://10.5281/zenodo.6038389. We assigned the initial version number 1.0.0 in conjunction with making the zenodo deposit. The code is written in python 3 MadHitter requires either of the publicly available packages SCIP (https://www.scipopt.org/) or Gurobi (https://www.gurobi.com) to solve integer linear programs to optimality. All MadHitter analyses using SCIP used either version 6.0.1 or version 6.0.2 of SCIP and the SCIP version does not affect the results. All MadHitter analyses using Gurobi were done using version 9.0.0 or 9.1.0.

For manuscripts utilizing custom algorithms or software that are central to the research but not yet described in published literature, software must be made available to editors and reviewers. We strongly encourage code deposition in a community repository (e.g. GitHub). See the Nature Research guidelines for submitting code & software for further information.

Data

Policy information about availability of data

All manuscripts must include a data availability statement. This statement should provide the following information, where applicable:

- Accession codes, unique identifiers, or web links for publicly available datasets
- A list of figures that have associated raw data
- A description of any restrictions on data availability

All our analyses are based on data publicly available in GEO or ArrayExpress. The lightly processed and reformatted input files are provided at https://ftp.ncbi.nlm.nih.gov/catSMA/MadHitter_data

Field-specific reporting

Please select the one below that is the best fit for your research. If you are not sure, read the appropriate sections before making your selection.

[x] Life sciences [ ] Behavioural & social sciences [ ] Ecological, evolutionary & environmental sciences

For a reference copy of the document with all sections, see nature.com/documents/nr-reporting-summary-flat.pdf

Life sciences study design

All studies must disclose on these points even when the disclosure is negative.

Sample size	We used all single-cell data sets we could find as of January 2019 that had sufficient number of cancer and non-cancer cells and at least 3 patients. Exclusion of some data sets that had only cancer cells is explained in the paper. For computational experiments, we generally sampled 500, 250, or 100 cells depending on the number available. Robustness of this assumption is assessed in Figure 2B and some supplementary analyses.
Data exclusions	Data sets with only cancer cells were excluded. Patients for which sampling typically selected ~10 or fewer cancer cells or non-cancer cells were excluded. Patients for whom there was not matching cancer and non-cancer data were excluded.
Replication	We did two types of replication. First, many analyses were done by sampling 20 replicates from the input data to arrive at each data point. Second, we ran some analyses using both SCIP and Gurobi libraries (see Data Analysis passage above). All attempts at replication were successful.
Randomization	In the standard meaning of randomization, this is not applicable because we are not testing treatements or testing some hypothesis between multiple conditions. We did use random (down)sampling of the input data to generate 20 replicates for most of our analyses, but this sampling is not about assigning patients to groups. We also sampled subsets of patients in one set of analyses to estimate the effect of patient cohort size on our figures of merit.
Blinding	Not applicable because all input data were anonymized to begin with and we are not making any assertions about what may have happened when patients were treated.

Reporting for specific materials, systems and methods

We require information from authors about some types of materials, experimental systems and methods used in many studies. Here, indicate whether each material, system or method listed is relevant to your study. If you are not sure if a list item applies to your research, read the appropriate section before selecting a response.

Materials & experimental systems

n/a	Involved in the study	
[x]	[ ]	Antibodies
[x]	[ ]	Eukaryotic cell lines
[x]	[ ]	Palaeontology and archaeology
[x]	[ ]	Animals and other organisms
[x]	[ ]	Human research participants
[x]	[ ]	Clinical data
[x]	[ ]	Dual use research of concern

Methods

n/a	Involved in the study	
[x]	[ ]	ChIP-seq
[x]	[ ]	Flow cytometry
[x]	[ ]	MRI-based neuroimaging
